# The Large-Scale Physical Model Tests of the Passing Ship Effect on a Ship Moored at the Solid-Type Berth

**DOI:** 10.3390/s22030868

**Published:** 2022-01-24

**Authors:** Teresa Abramowicz-Gerigk, Zbigniew Burciu, Tomasz Jaworski, Jacek Nowicki

**Affiliations:** 1 Faculty of Navigation, Gdynia Maritime University, 81-225 Gdynia, Poland; z.burciu@wn.umg.edu.pl; 2 Foundation for Safety of Navigation and Environment Protection, 14-200 Ilawa, Poland; tomaszj@portilawa.com (T.J.); director@portilawa.com (J.N.)

**Keywords:** passing ship, moored ship, large-scale ship model, physical model tests

## Abstract

The paper presents experimental research on hydrodynamic forces generated on a ship moored at a long quay wall, modeling the solid-type berth, by a passing ship. The proper prediction of interactions between the moored and passing ships is important for design and operational purposes. The results of the presented parametric study are presented as a space-time series of the forces as the functions of passing ship velocity and transverse separation distance between the ships. The experimental test setup constructed on the lake and the large scale of the manned physical ship models enabled a simulation of the real maneuvering situation. The force measurements were taken on the moored ship model fixed to the pantographs rigidly attached to the wall. The twin pantographs were attached to force sensors on the deck of the model by a system of movable joints, enabling the measurement of surge and sway forces. The presented method was verified based on available experimental and numerical data, showing a good agreement with the results.

## 1. Introduction

The list of variables used in the paper is presented in Table 1. 

The hydrodynamic forces generated on the ship moored at a pier by the passing ship are related to ship characteristics, berth construction and waterway geometry, separation distance between the ships, velocity of the passing ship, mooring and fendering system characteristics and hydrometeorological conditions. Their effects are not independent and may significantly change with other parameter changes. Flory et al. [1] have proposed a neural network analysis of passing ship forces, including all the influencing parameters. 

The proper prediction of interactions between moored and passing vessels is important for the design of reliable mooring and fendering systems, traffic safety analysis [2] and the design of controllers for autonomous vessels [3,4].

The motions of the moored ship generated by a passing vessel can interrupt loading operations [5,6]. For example, the maximum allowable motion amplitudes for ferries and ro–ro vessels during loading operations using ramps are 0.1 m for both sway and surge, and for tankers they can be about ten times bigger due to the use of flexible hoses [7]. 

The empirical, experimental and numerical methods [8,9,10] are used to determine the passing ship’s generated forces. 

Empirical methods may include hydrodynamic interaction and environmental forces from wind, current, waves and shallow-water effects [11]. Vesudeven and Nallayarasu [12] presented a comprehensive review of existing methods developed for passing ship effect prediction. They verified the applicability of each method to certain conditions, including the combined effect of loads generated by a passing vessel and environmental forces.

Most of the experiments described in the available literature were carried out in towing tanks in deep-water conditions, without modeling a long quay wall, or with the modeled quay wall but in shallow-water conditions. The geometrical scales used in towing tank tests were 1:32 [13], 1:60 [14], 1:68 [15], 1:100 [16,17], or 1:135 [18]. 

The small geometrical scale entails not only a larger-scale effect, but also flow blockage, water cushion formation or flow separation phenomena different from ones observed in real scale. The hydrodynamic forces were mainly measured using specially designed experimental test setups.

Kriebel et al. [18] presented the model test results of 1.524 m long models of Panamax-size vessels in 1:135 geometrical scale with Series 60 hull forms. The mooring loads and sway and surge forces were measured by the fore-and-aft force gauges attached to the rigid frame using longitudinal and transverse carbon-fiber rods, allowing the model to heave, pitch and roll. Three force gauges of 0.89 N capacity and about 0.022 N resolution connected to the rods were placed on the rigid aluminum frame. The passing ship attached to the towing carriage was free to squat and trim. The influence of the water depth-to-draft ratio and separation distance were studied. The long quay wall was not modeled in these experiments. 

Swiegers [17] carried out the measurements of mooring forces using a 1:100 scale model of a bulk carrier and container ship. The bulk carrier model was moored at the long tight wall to a fixed frame in the setup configuration, similar to the one proposed by Kriebel [18]. The hydrodynamic forces were measured at a sampling rate of 50 Hz with force transducers attached to the frame. The passing container ship model free to heave, roll and pitch was towed on a parallel course by a trolley along a rail. 

**Figure 1:**
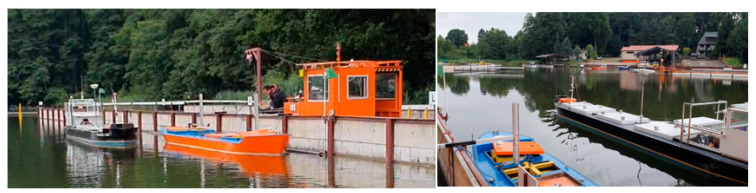
The testing area at the Ship Handling Research and Training Center on Lake Silm.

The research presented in this paper was carried out at the Ship Handling Research and Training Center, belonging to the Foundation for Safety of Navigation and Environmental Protection located on Lake Silm near Ilawa (Figure 1).

The objective of this study was the physical modeling of a real maneuvering situation. The conditions corresponding to the real port and waterway operation were modeled using the manned model of a passing ship moving on a straight course, parallel to the long quay wall, using its own propulsion and steering devices. This is different from the captive model tests carried out in towing tanks, with the passing ship towed on a straight course by the towing carriage, usually free to trim, pitch and roll. 

The research focused on measurements of the passing ship’s generated forces using large-scale manned physical 1:24 scale models of Panamax-size bulk carriers. 

The results obtained for the large physical models can be used to determine the forces generated on real ships and to verify the results of numerical simulations. The larger scale allowed for a more precise measurement of forces in the range of 100 N and for reducing the scale effect. The modeled navigational procedure in deep-water conditions presented in the paper allowed for a better observation of the separation distance and passing ship velocity influence on the hydrodynamic forces. 

Huang and Chen [9] reported that radiation forces generated in response to vessel motions while it is moderately moored can reduce the force generated on a moored ship by 50%. Measurements of forces on a stationary model in surge, sway and yaw directions enabled the elimination of the influence of radiation forces and the measurement of hydrodynamic forces not reduced by them. The construction of the experimental test setup also allowed for the elimination of the influence of mooring and fendering system characteristics on the measured forces.

The assumptions adopted in this study allowed eliminating these forces, as well as the dependence of the measured forces on mooring and fendering system characteristics. 

It has been proved by Swiegers [17] that for a ship sailing parallel to the moored ship, regardless of the direction of passing from the bow or stern, the forces generated during the passage of the ship are similar Therefore, in the presented research, the parallel configuration of ships most often used in operational practice, and the direction passing ship movement from the stern to the bow of the moored ship were adopted. 

In the assumed configuration with ships of similar dimensions, the most important operational parameters were passing ship velocity and transverse separation distance. These parameters decide on the impact of the moored ship on fenders and mooring lines, as their limits are used as risk reduction options of mooring system failure. 

The paper describes in detail the experimental test setup, ship model characteristics and instrumentation. 

## 2. Materials and Methods

### 2.1. Measurement of Interaction Forces Generated on the Moored Ship Model

The experimental test setup was located at the rectilinear part of the training area, modeling a canal constructed of two vertical tight walls. The moored ship model was positioned outside the canal and fixed to the canal wall by bow and aft pantographs, enabling the measurements of surge and sway forces without the influence of model trim, roll and pitch. The large open area at starboard side of the moored model allowed the undisturbed movement of the passing ship model on the course parallel to the moored ship. This manned model was moving at the assumed transverse distance with the constant speed in the leading line. The leading mark allowed keeping the straight course in the assumed separation distance. 

The long tight wall, vertically extending to the bottom of the lake, was used to model the solid-type berth. The deep-water conditions at the tested velocity range were ensured by the depth-to-draft ratio of h/T = 4.

**Figure 2:**
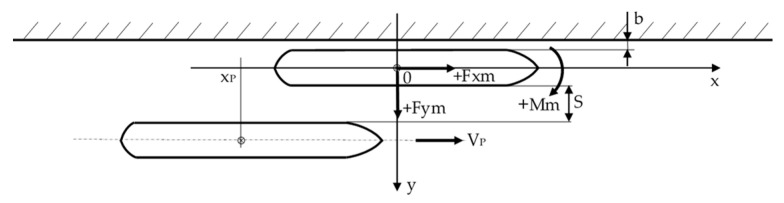
The scheme of the experimental test setup and coordination system 0xy related to the moored ship adopted for measurements. x_P_—position of the amidships of the passing ship model, Fxm, Fym—measured surge and sway forces, Mm—yaw moment, b—distance between the side of the moored ship model and quay wall, v_P_—velocity of the passing ship model, S—separation distance.

The scheme of the experimental test setup and clockwise coordination system, with the origin 0 located at amidships of the moored ship and *z*-axis pointing out of the page adopted for measurements, is presented in Figure 2. The ship motions presented in the paper are consistent with this system.

The distance b between the side of the moored model and the wall was equal to 0.3 m. The main dimensions of the models used in the experiment are presented in Table 2. 

### 2.2. Measuring Sensors and Procedure

**Figure 3:**
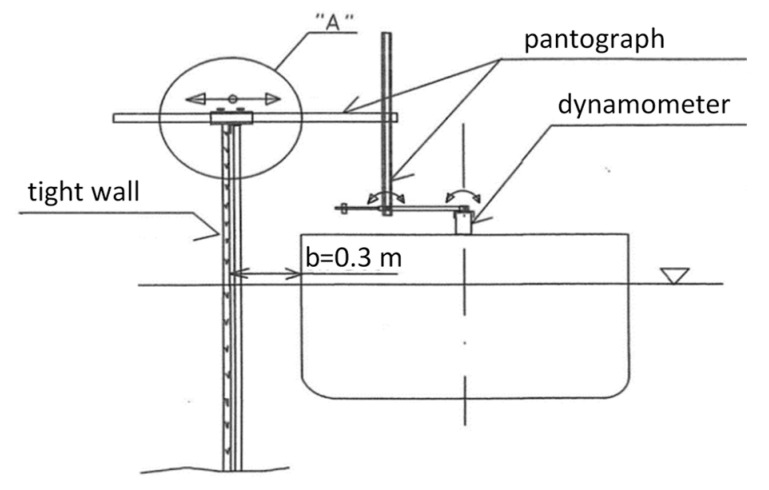
Scheme of the experimental test setup—side view of the supporting construction.

The moored model fixed to the supporting construction in form of two pantographs was free to heave, roll and pitch. The pantographs holding the moored model in a fixed position were attached to the dynamometers placed on the moored ship deck by a system of movable joints. The second end of each pantograph was fixed to the wall. The side view of the supporting construction with force sensor-dynamometer are presented in Figure 3. 

**Figure 4:**
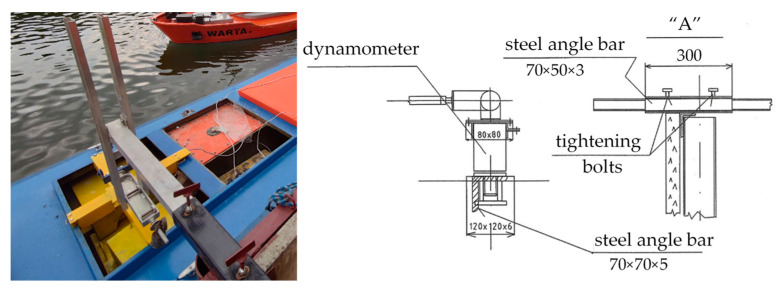
Scheme of the experimental test setup—details of the supporting construction.

The rigid, aluminum structure ensured the measurement of the forces without interference from the mechanical system. The view and scheme of the joints, with the detail “A” marked in Figure 3, are presented in Figure 4. 

**Figure 5:**
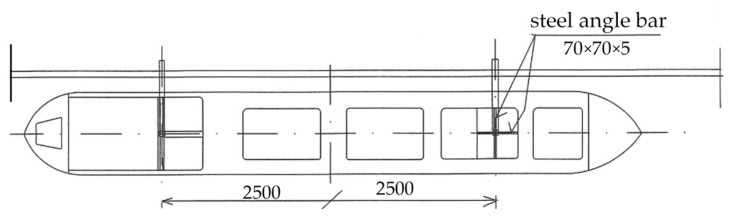
Scheme of the experimental test setup—top view.

The top view of the supporting structure is presented in Figure 5.

Force sensors (SPAIS FT-5953) with dedicated amplifier blocks (SPAIS AT-5253) were used to measure the forces.The sensors allowed for measuring the force in two axes in 0xy system and two directions within the range of ±200 kN. The amplifier blocks converted electrical signals from the sensors into analog voltage signal proportional to the applied force. The dynamometers were mounted at the deck level of the ship model, along its longitudinal axis x. They were moved symmetrically in relation to the amidships by a distance of 5 m, as shown in Figure 5. The accuracy of the dynamometers used, according to the PN-EN ISO 7500-1 standard, is 1%, which is sufficient accuracy in the presented experiment.

The voltage signals were read using the ADAM-6024 analog-to-digital converter from Advantech with 16-bit resolution on each channel. The processed signal acquisition was carried out with specially prepared, dedicated software, which, in addition to reading and recording the voltage values from the x and y channels of each sensor, also recorded the sampling time to enable subsequent data processing. 

**Figure 6:**
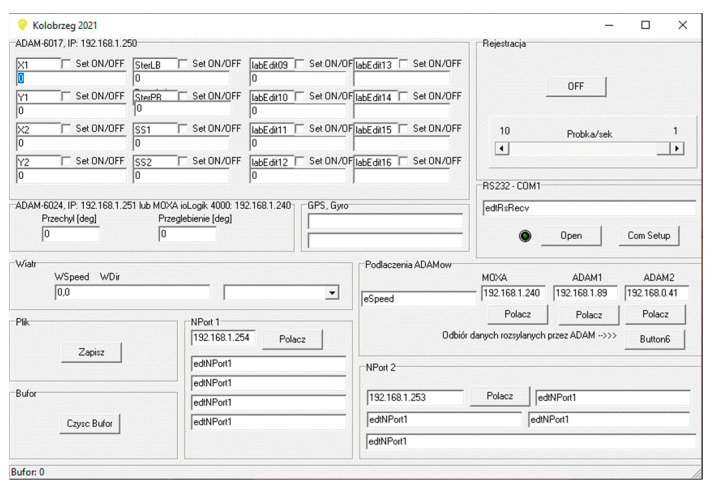
Window of data logging program.

The window of the data logging program is presented in Figure 6.

In order to ensure fast and error-free data exchange, the Modbus/TCP communication standard was used for data transmission between the transmitter and computer. Time-based recording also enables synchronization of the recorded data with data containing trajectories of the passing ship model, which were recorded in a separate system.

The position and speed of the passing ship model were measured using the Leica GS10 GPS receiver, operating in RTK mode. It provides position measurement with an accuracy of ±0.01 m. The measurement of a heading angle was carried out by the Anschutz STD22 gyrocompass, commonly used on real ships. The data from the devices were transmitted and recorded by a telemetry system used on the training models of Ship Handling Research and Training Center.

The program of tests consisted of surge and sway force measurements under various separation distances and different passing velocities. The used values of non-dimensional separation distance s Equation (1), Froude number Equation (2) and real-scale ship velocity, calculated according to the Froude principle of similarity Equation (3), are presented in Table 3.

(1) $$s\;=\;S/B_{m}$$

(2) $$\mathrm{Fn}=v_{P}/\sqrt{{\mathrm{gL}}_{\mathrm{WLP}\;}}$$

(3) $$v=v_{P}\sqrt{\lambda }$$

where s is the non-dimensional separation distance; S (m) is the separation distance; B_m_ (m) is the moored model breadth; v_P_ (m/s) is the passing ship model velocity; g (m/s^2^) is the gravitational constant; L_WLP_ (m) is the passing ship model length at waterline; and 1:λ is the geometrical model scale.

## 3. Results

**Figure 7:**
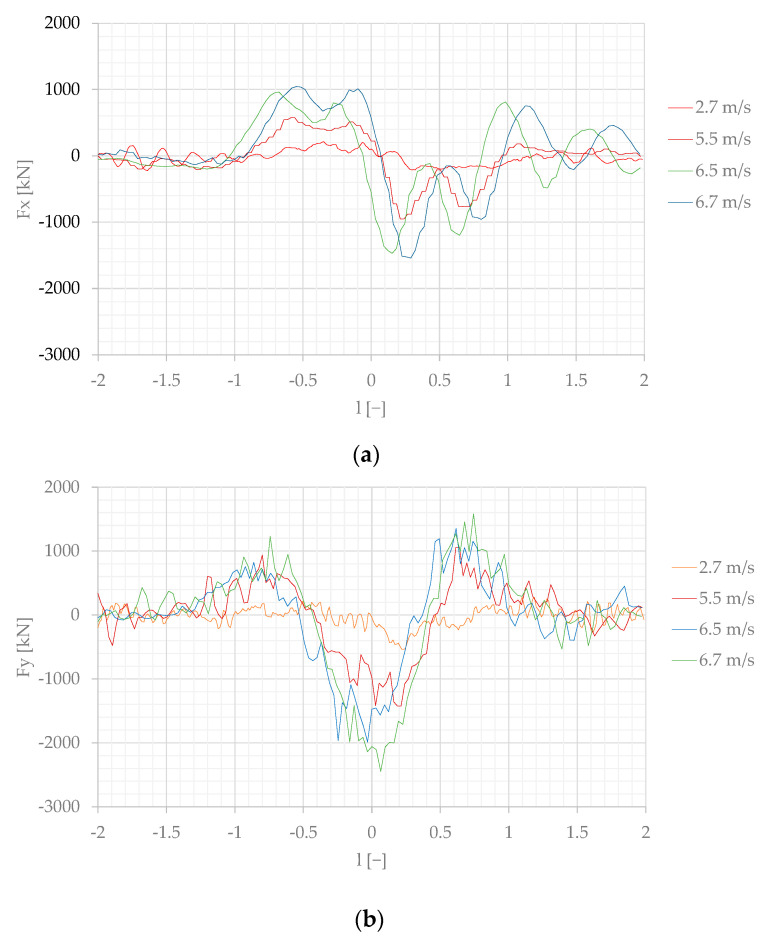

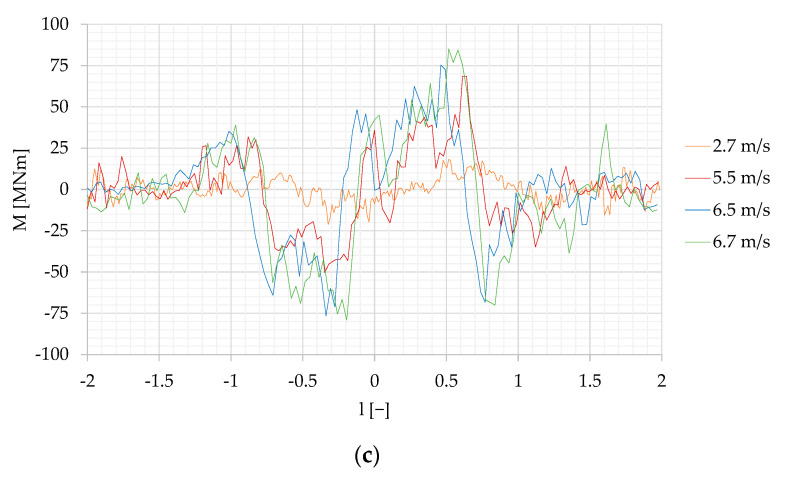
Forces measured on the moored ship at the separation distance s = 1, converted to the real scale: (**a**) longitudinal force Fx; (**b**) transverse force Fy; (**c**) yaw moment M.

**Figure 8:**
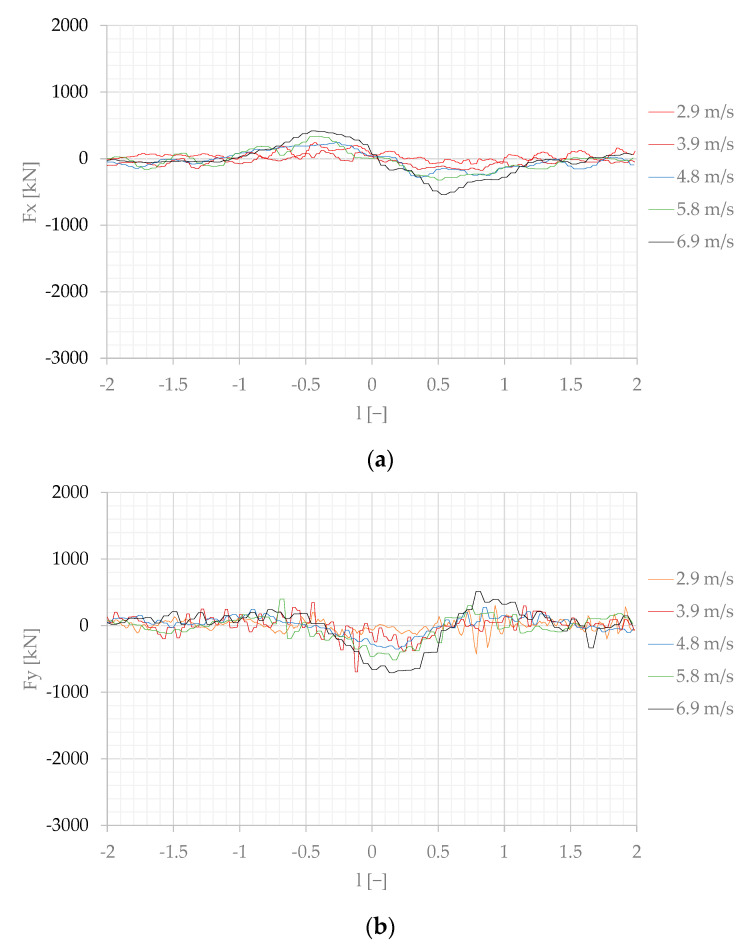

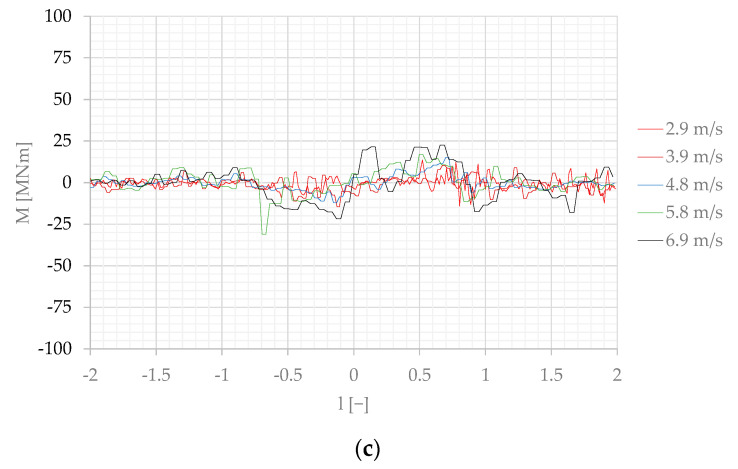
Forces measured on the moored ship at the separation distance s = 2, converted to the real scale: (**a**) longitudinal force Fx; (**b**) transverse force Fy; (**c**) yaw moment M.

**Figure 9:**
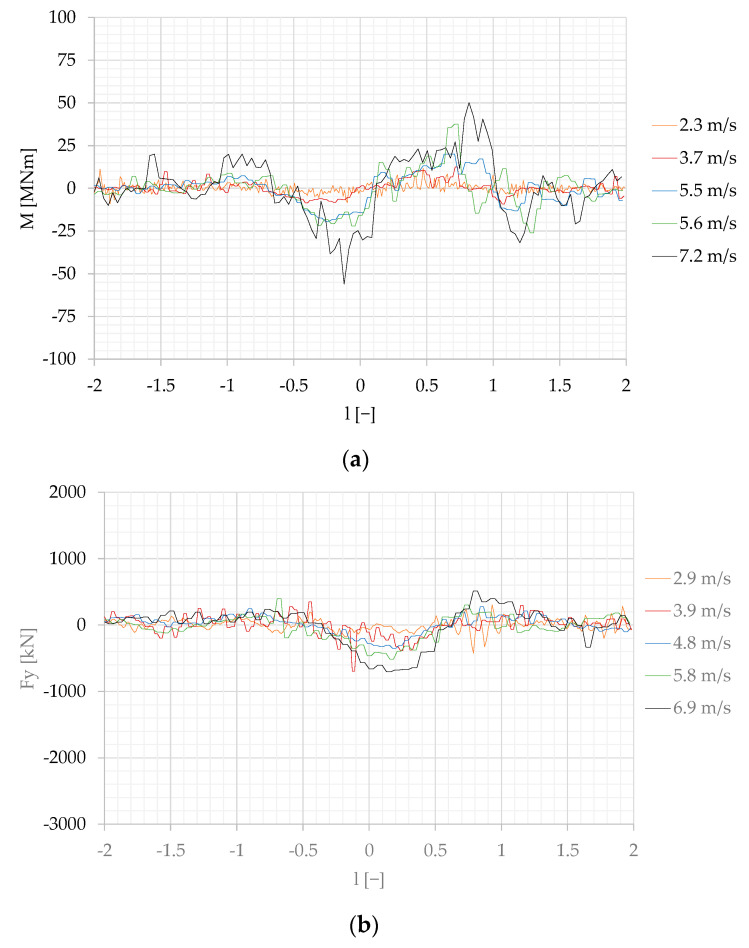

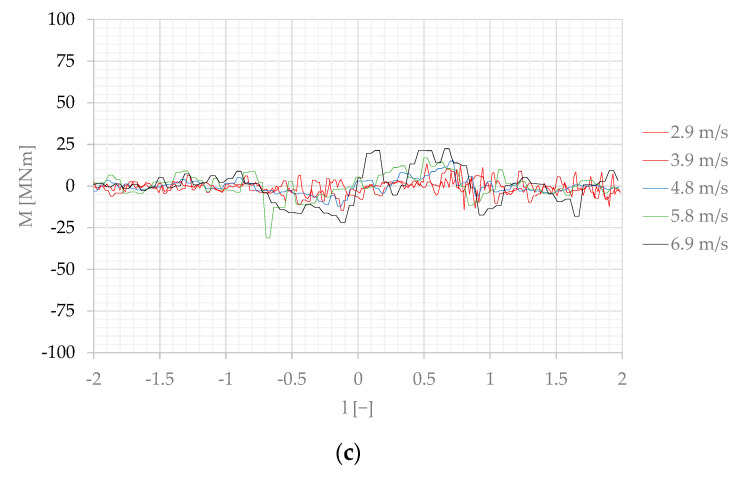
Forces measured on the moored ship at the separation distance s = 3, converted to the real scale: (**a**) longitudinal force Fx; (**b**) transverse force Fy; (**c**) yaw moment M.

The forces measured on the moored model converted to the real scale, according to the Froude law of similarity Equation (4), are presented in Figure 7, Figure 8 and Figure 9.

$$\mathrm{Fx}=\mathrm{Fxm}\cdot \lambda^{3}$$

(4) $$\mathrm{Fy}=\mathrm{Fym}\cdot \lambda^{3}$$

$$M=\mathrm{Mm}\cdot \lambda^{4}$$

where 1:λ is the geometrical model scale; Fx (N), Fy (N) and M (Nm) are surge and sway forces and yaw moment in real scale; and Fxm (N), Fym (N) and Mm (Nm) are, respectively, surge and sway forces and yawing moment measured on the moored model.

The non-dimensional position of the passing ship l is defined as the distance between both vessels amidships, defined by Equation (5):

(5) $$l=\frac{x_{P}}{L_{\mathrm{WLP}}}=\left(t\cdot v-2L_{\mathrm{WLP}}\right)/L_{\mathrm{WLP}}$$

where x_p_ x_P_ (m) is the position of the passing ship model amidships; L_WLP_ (m) is the length at waterline of the moored model; and t (s) is the time of passing maneuver. The initial time of each trial t = 0 is related to the initial passing ship position at x_P_ = −2$L_{\mathrm{WLP}}$.

**Figure 10:**
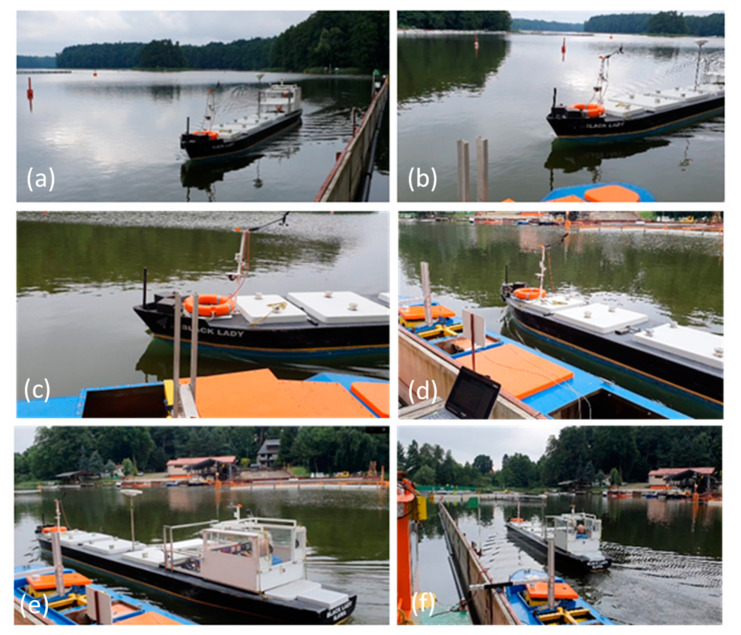
Wave system generated by the passing ship model at s = 1, Fn = 0.145: (**a**) l = −1.5; (**b**) l = −1; (**c**) l = −0.75; (**d**) l = −025; (**e**) l = 0.25; (**f**) l = 1.

**Figure 11:**
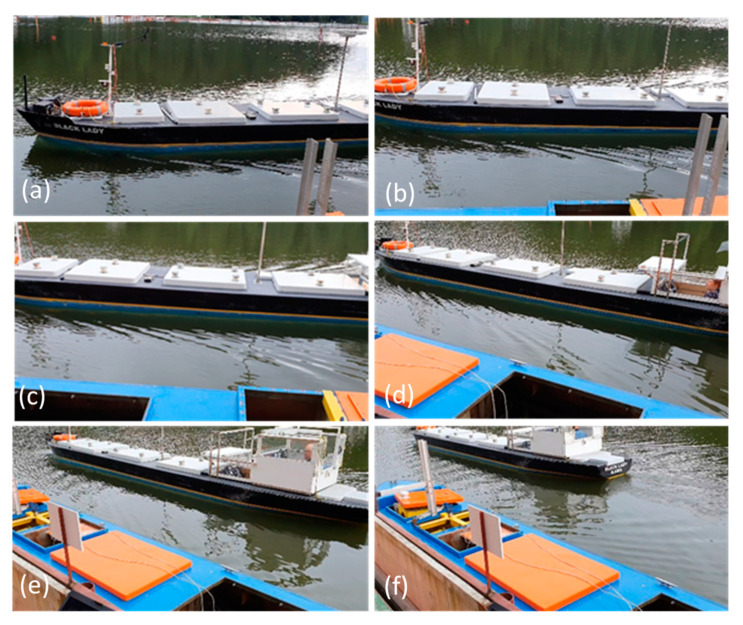
Wave system generated by the passing ship model s = 2, Fn = 0.155: (**a**) l = −0.5; (**b**) l = −0.25; (**c**) l = 0; (**d**) l = 5; (**e**) l = 0.25; (**f**) l = 0.75.

The waves generated at s = 1 and s = 2 are presented in Figure 10 and Figure 11. The ship-induced wave trains are often decomposed into two components. The first component is the primary wave system, with wave crests generated in the bow and stern high-pressure areas, and wave through along the ship length called drawdown. At low ship speeds, the primary system of long period and large length waves has the biggest influence on the moored ship. The primary waves are superimposed on the secondary system of short period and small length waves, generated by the disturbances at bow and stern. The secondary system includes two sets of diverging waves and transverse waves. Secondary waves with much shorter periods can carry high energy and have erosive potential in the shallow bank zone [19,20].

The first influence of the passing ship sailing at the shortest separation distance s = 1 was observed at l = −2 due to the bow pressure field generated by the passing vessel. Then, a significant increase in the surge force started at l = −1, sway force at l = −1.5, and it was changed to the opposite value at l = −0.25 to the highest values of 1400–1600 kN between l = 0 and l = 0.25, at passing speeds of 6.5 m/s and 6.7 m/s.

The effect of the separation distance is shown in Figure 8 and Figure 9, where the highest values at s = 1 and s = 2 are, respectively, twice and four times smaller than at s = 1.

The maximum negative surge force at s = 2 appears at l = 0.5 to 0.7. This phenomenon is related to the moment of reflected wave formation on the moored vessel due to the changes in velocity field. Similar phenomena, also at bigger separation distances and higher passing velocities, were observed by Nam and Park [21] in their numerical study.

At s = 1, when the passing ship stern passed the bow of the moored ship (l = 1), the strong influence of the stern wave and the influence of the wave system reflected from the quay wall, shown in Figure 11f, are the reasons for a surge force equal to 0.8 of the maximum positive surge force generated by the bow wave system at l = −0.7. The wave system reflected from the quay wall was not observed at longer passing distances (Figure 7, Figure 8 and Figure 11f). 

The beginning of reflected waves formation and developed wave system can be seen in Figure 11e,f. 

**Figure 12:**
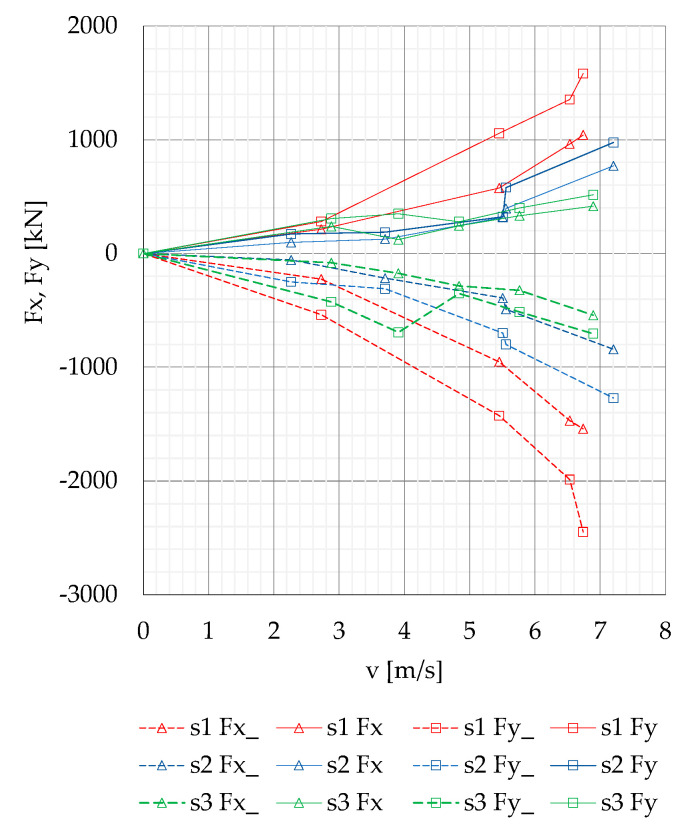
Maximum positive and negative surge and sway forces Fx, Fx_, Fy and Fy_ versus passing ship velocity v, for the separation distances s = 1, 2 and 3.

The variability of maximum positive and negative surge and sway forces Fx, Fx_, Fy and Fy_ measured on the moored model depending on passing ship velocity at separation distances s = 1 and s = 2 presented in Figure 12, is smooth. Analyzing the forces generated on the moored vessel without a quay wall influence, Kriebel et al. [18] determined a simple quadratic dependence. A similar dependence is presented in Figure 12.

## 4. Discussion

**Figure 13:**
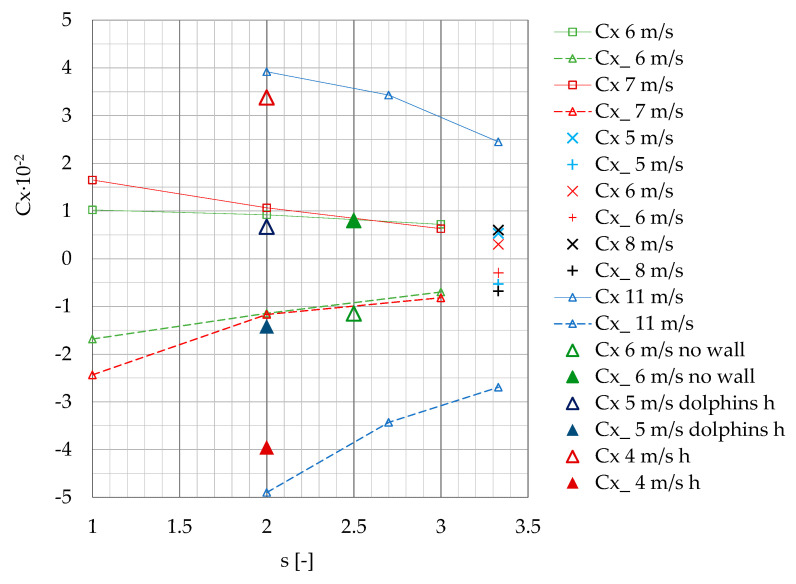
Cx and Cx_ coefficients of maximum positive and negative values of the measured Fx forces versus separation distance s at v = 6 m/s and 7 m/s (Fn = 0.12 and Fn = 0.15) and gap distance 0.2 B, compared with the published results: 5 m/s, 6 m/s, 8 m/s, 11 m/s: v = 5, 6, 8, 11 m/s (Fn = 0.15, 0.2, 0.25, 0.35), gap distance b = 0.3 B [21]; 6 m/s no wall: v = 6 m/s (Fn = 0.14), no influence of a long quay wall [18]; 5 m/s dolphins h: v = 5 m/s (Fn = 0.07), quay wall with dolphins, shallow water [24]; 4 m/s h: v = 4 m/s (Fn = 0.08), shallow water [17].

**Figure 14:**
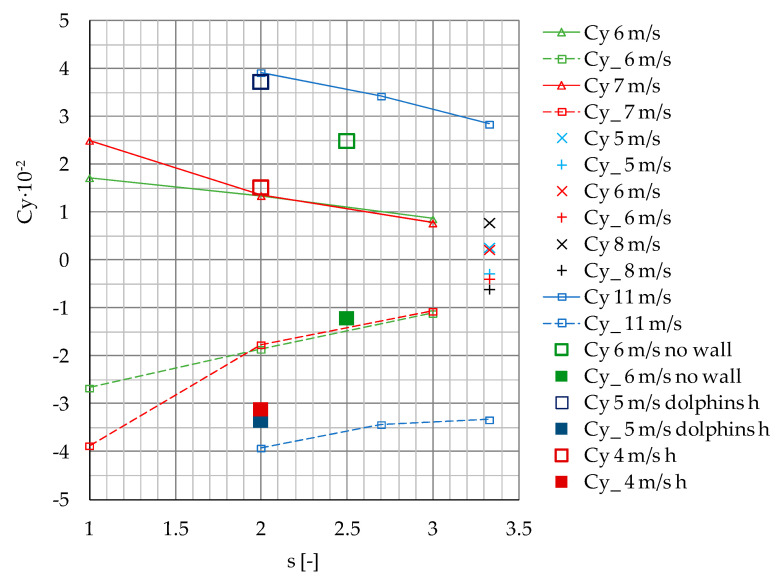
Cy, Cy_ coefficients of maximum positive and negative values of the measured Fy forces versus separation distance s at v = 6 m/s and 7 m/s (Fn = 0.12 and Fn = 0.15) and gap distance 0.2 B, compared with the published results: 5 m/s, 6 m/s, 8 m/s, 11 m/s: v = 5, 6, 8, 11 m/s (Fn = 0.15, 0.2, 0.25, 0.35), gap distance b = 0.3 B [21]; 6 m/s no wall: v = 6 m/s (Fn = 0.14), no influence of a long quay wall [18]; 5 m/s dolphins h: v = 5 m/s (Fn = 0.07), quay wall with dolphins, shallow water [24]; 4 m/s h: v = 4 m/s (Fn = 0.08), shallow water [17].

The results of the presented model tests were compared to the available experimental data in smaller model scales [18] and numerical computations [21,22], both assuming deep-water conditions (Figure 13 and Figure 14). The data available in the literature, including the quay wall effect, are mainly available for small depth-to-draft ratios [23]. The significant shallow-water effect is illustrated in Figure 13, on the example of model test results of a container ship passing a moored bulk carrier in 1:100 model scale [17]. 

To compare the results presented by different authors, the coefficients of hydrodynamic forces related to the passing ship speed, moored ship length and draft are defined as follows [21,22] Equations (6) and (7):

(6) $$\mathrm{Cx}=\mathrm{Fx}/\left(0.5\cdot {\rho v}^{2}\mathrm{LT}\right)$$

(7) $$\mathrm{Cy}=\mathrm{Fy}/\left(0.5\cdot {\rho v}^{2}\mathrm{LT}\right)$$

where Cx_,_ Cy are the coefficients for surge force and sway force respectively; ρ (kg/m^3^) is water density; v (m/s) is the passing ship velocity; T (m) is the moored ship draft; and L (m) is the length of the moored ship. 

The coefficients of the maximum positive and negative values of Fx and Fy forces measured at v = 6 m/s and v = 7 m/s (Fn = 0.12 and Fn = 0.15), and gap distance 0.2 B, tend to be in line with the results obtained by Nam and Park et al. [21] at larger separation distance s = 3.33, larger gap distance b = 0.3 B and passing ship velocities v = 5 m/s, v = 6 m/s and v= 8 m/s (Fn = 0.15, Fn = 0.20 and Fn = 0.25). 

Based on previous experiments, it was confirmed that, at low speeds, the interaction forces increase with the square of passing ship velocity [21]. The coefficients obtained in this study for s = 2 and 3 and passing ship velocity v = 6 m/s are three to four times smaller than the coefficients obtained by Nam and Park [21] at passing ship velocity equal to 11 m/s (Fn = 0.35). The influence of the passing distance on sway and surge force coefficients exhibits similar trends in both cases.

The results of model scale measurements obtained by Kriebel [18] for the ship moored at a short pier, without the influence of a long quay wall, at passing ship velocity 6 m/s (Fn = 0.16), show similar values of surge force coefficients and 2.5 greater maximum sway force coefficient than the corresponding coefficients obtained in this study. 

There is a similar dependence between the surge force coefficients presented by Fenical [24], obtained for passing and moored ULCSs (Ultra Large Container Ships) in real scale, in shallow-water conditions and v = 5 m/s. The ship was moored to the long berth with dolphins, ending close to the moored ship bow. There was no influence of the tight wall. The maximum and minimum sway force coefficients were respectively 2.5 and 1.9 times greater than the corresponding coefficients obtained in this study. 

The results of model tests in shallow-water conditions at water depth-to-draft ratio of h/T = 1.2 and slower passing speed v = 4 m/s presented by Swiegers in his Test No. 16 [17] show 3.5- and 4-times higher values of the maximum and minimum surge force coefficients, respectively, than the values measured at v = 6 m/s in the present study. The minimum sway force coefficient was 1.8 times greater; however, the maximum sway force coefficient was only a little greater than the coefficients obtained in deep water at higher speeds.

## 5. Conclusions

The presented experiments were carried out at Reynolds numbers from 2.4∙10^7^ to 6.1∙10^7^; therefore, the scale effects related to the viscosity can be neglected. Previous experiments carried out using large-scale models in deep- and shallow-water conditions [25] confirmed the strong influence of model scale on the flow-generated hydrodynamic forces in shallow water. The effects of flow blockage, water cushion formation and flow separation dependent on model scale, are more pronounced in shallow-water conditions than in deep water. The next part of the studies on the passing ship’s generated forces will be presented for shallow-water conditions.

The main conclusions are as follows:

1. The results presented in Figure 13 and Figure 14 confirmed the general dependence of the forces in shallow-water conditions. The forces generated in shallow water at much smaller Froude numbers Fn = 0.07 [17] and Fn = 0.08 [25] were not less than forces generated at Fn = 0.12 and Fn = 0.15 in deep water.
2. The effect of moored ship’s earlier reaction to a ship passing at a slower speed than that of a vessel passing at higher speed had been noticed by Huang and Chen [9], who explained this phenomenon as an earlier attainment of the maximum value by force at lower speed than at higher speed. It is more pronounced at shorter passing distances at s = 1 and s = 2 (Figure 7a or Figure 8a).
3. The main objective of this work was to expand the experimental database for mooring loads generated by a passing ship; therefore, in this study, raw measurements are presented.

The comparative results of model tests and numerical simulations were available in filtered form, without higher frequency oscillations, which have a small effect on the moored ship. This small effect is caused by high inertia of the moored ship [17,18,21]. Swiegers [17] and Kriebel [18] determined the minimum and maximum surge and sway forces using the smoothed time history of passing ship forces. Kriebel [18], in the analysis of results of model tests in 1:135 scale, used a low-pass filter with 2 Hz cut-off frequency, isolating low frequency loads.
Raw data are overestimated in relation to filtered data. However, due to different experimental conditions, e.g., large-scale and self-propelled manned model, it was only possible to show that the obtained measurements were close to other results and had a similar trend of changes, depending on the influencing parameters.

4. The general conclusions with respect to passing ship forces are that they increase with increasing passing velocity and with decreasing separation distance [13,17,21,22].
5. Passing ship forces increase significantly if the passing ship sails at a drift angle [13]. Changing speed and course of a ship changes the phase and magnitude of the fluid forces. In the presented research, the reactions of the passing self-propelled manned ship model were compensated by the corrections of the propeller thrust and rudder angle, which enabled the maintenance of a constant course and constant speed in all tests.

## Figures and Tables

**Table 1:** List of variables used in the paper.

Parameter	Description
B	moored ship breadth
b	gap distance between moored ship side and wall
D	ship draft
Fx, Fy	surge and sway forces
h	water depth
L	overall moored ship length
L_WL_	passing ship length at waterline
M	yaw moment
_m_	subscript for the moored ship model
_P_	subscript for passing ship model
S	separation distance between moored and passing ship
s	non-dimensional separation distance
T	moored ship draft
v	passing ship speed
x_P_	position of the midship of the passing ship model
1:λ	geometrical model scale

**Table 2:** Main dimensions of models used in the experiment.

Ship Model	L	L_WL_	B	D
	m	m	m	m
Moored ship model	9.49	9.0 m	1.26 m	0.51
Passing ship model	9.17	9.3 m	1.34 m	0.52

**Table 3:** Test parameters.

s = 1			s = 2			s = 3		
Fn	v_P_	v	Fn	v_P_	v	Fn	v_P_	v
	m/s	m/s		m/s	m/s		m/s	m/s
0.059 0.117 0.140 0.145	0.56 1.11 1.33 1.38	2.7 5.5 6.5 6.7 7.2	0.049 0.080 0.119 0.155 0.199	0.46 0.76 1.12 1.13 1.47	2.3 3.7 5.5 5.6 7.2	0.062 0.084 0.104 0.124 0.148	0.59 0.80 0.99 1.18 1.41	2.9 3.9 4.8 5.8 6.9
